# Reduced neutralization of SARS-CoV-2 B.1.1.7 variant by convalescent and vaccine sera

**DOI:** 10.1016/j.cell.2021.02.033

**Published:** 2021-04-15

**Authors:** Piyada Supasa, Daming Zhou, Wanwisa Dejnirattisai, Chang Liu, Alexander J. Mentzer, Helen M. Ginn, Yuguang Zhao, Helen M.E. Duyvesteyn, Rungtiwa Nutalai, Aekkachai Tuekprakhon, Beibei Wang, Guido C. Paesen, Jose Slon-Campos, César López-Camacho, Bassam Hallis, Naomi Coombes, Kevin R. Bewley, Sue Charlton, Thomas S. Walter, Eleanor Barnes, Susanna J. Dunachie, Donal Skelly, Sheila F. Lumley, Natalie Baker, Imam Shaik, Holly E. Humphries, Kerry Godwin, Nick Gent, Alex Sienkiewicz, Christina Dold, Robert Levin, Tao Dong, Andrew J. Pollard, Julian C. Knight, Paul Klenerman, Derrick Crook, Teresa Lambe, Elizabeth Clutterbuck, Sagida Bibi, Amy Flaxman, Mustapha Bittaye, Sandra Belij-Rammerstorfer, Sarah Gilbert, David R. Hall, Mark A. Williams, Neil G. Paterson, William James, Miles W. Carroll, Elizabeth E. Fry, Juthathip Mongkolsapaya, Jingshan Ren, David I. Stuart, Gavin R. Screaton

**Affiliations:** 1 Wellcome Centre for Human Genetics, Nuffield Department of Medicine, University of Oxford, Oxford, UK; 2 Division of Structural Biology, Nuffield Department of Medicine, University of Oxford, The Wellcome Centre for Human Genetics, Oxford, UK; 3 Chinese Academy of Medical Science (CAMS) Oxford Institute (COI), University of Oxford, Oxford, UK; 4 Oxford University Hospitals NHS Foundation Trust, Oxford, UK; 5 Diamond Light Source Ltd, Harwell Science & Innovation Campus, Didcot, UK; 6 National Infection Service, Public Health England (PHE), Porton Down, Salisbury, UK; 7 Peter Medawar Building for Pathogen Research, Oxford, UK; 8 NIHR Oxford Biomedical Research Centre, Oxford, UK; 9 Translational Gastroenterology Unit, University of Oxford, Oxford, UK; 10 Centre For Tropical Medicine and Global Health, Nuffield Department of Medicine, University of Oxford, Oxford, UK; 11 Mahidol-Oxford Tropical Medicine Research Unit, Bangkok, Thailand; 12 Nuffield Department of Clinical Neurosciences, University of Oxford, Oxford, UK; 13 Nuffield Department of Medicine, University of Oxford, Oxford, UK; 14 Oxford Vaccine Group, Department of Paediatrics, University of Oxford, Oxford, UK; 15 Worthing Hospital, Worthing, UK; 16 MRC Human Immunology Unit, MRC Weatherall Institute of Molecular Medicine, Radcliffe Department of Medicine, University of Oxford, Oxford, UK; 17 Jenner Institute, Nuffield Department of Medicine, University of Oxford, Oxford, UK; 18 Sir William Dunn School of Pathology University of Oxford, Oxford, UK; 19 Siriraj Center of Research Excellence in Dengue & Emerging Pathogens, Dean Office for Research, Faculty of Medicine Siriraj Hospital, Mahidol University, Thailand; 20 Instruct-ERIC, Oxford House, Parkway Court, John Smith Drive, Oxford, UK

**Keywords:** SARS-CoV-2, B.1.1.7, Kent, variant, antibody, escape, neutralization, IGHV3-53

## Abstract

SARS-CoV-2 has caused over 2 million deaths in little over a year. Vaccines are being deployed at scale, aiming to generate responses against the virus spike. The scale of the pandemic and error-prone virus replication is leading to the appearance of mutant viruses and potentially escape from antibody responses. Variant B.1.1.7, now dominant in the UK, with increased transmission, harbors 9 amino acid changes in the spike, including N501Y in the ACE2 interacting surface. We examine the ability of B.1.1.7 to evade antibody responses elicited by natural SARS-CoV-2 infection or vaccination. We map the impact of N501Y by structure/function analysis of a large panel of well-characterized monoclonal antibodies. B.1.1.7 is harder to neutralize than parental virus, compromising neutralization by some members of a major class of public antibodies through light-chain contacts with residue 501. However, widespread escape from monoclonal antibodies or antibody responses generated by natural infection or vaccination was not observed.

## Highlights

• Original strain convalescent and vaccine sera show reduced B.1.1.7 neutralization
• N501Y enhances RBD: ACE2 binding affinity
• N501Y compromises neutralization by many antibodies with public V-region IGHV3-53
• No widespread escape by B.1.1.7 was observed

## Graphical abstract

**Figure:**
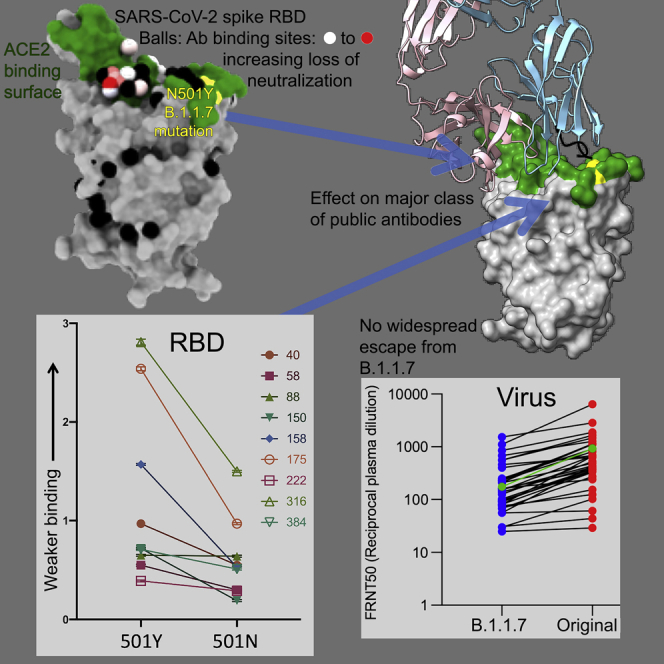

## Teaser

The SARS-CoV-2 B.1.1.7 variant is not neutralized as easily as the original form of the virus. Some public antibodies cannot neutralize B.1.1.7, due to altered light-chain contacts with residue 501. However, B.1.1.7 does not show widespread escape from monoclonal antibodies, natural antibody responses, or vaccines.

## Introduction

Since its first appearance in Wuhan in December 2019, SARS-CoV-2 rapidly spread around the world leading the WHO to declare a pandemic on March 11, 2020. Since then, drastic public health measures, including draconian lockdowns with severe economic cost, have been enacted to contain virus spread. Although initially successful at containing disease, many countries are now experiencing further waves of infection, coinciding with winter in the northern hemisphere, with infections in some countries outpacing those seen during the first wave (Kröger and Schlickeiser, 2021).

Huge strides have been made in the understanding of SARS-CoV-2 over the last year, which are exemplified by the licensing of several vaccines (in the UK those made by Pfizer-BioNtech, Moderna, and Oxford-AstraZeneca), which are being rolled out in massive global vaccination programs, with the aim to reach billions of individuals in 2021. Furthermore, Janssen and Novavax have recently reported results showing good efficacy and also report efficacy against the UK B.1.1.7 strain (https://blogs.sciencemag.org/pipeline/archives/2021/01/29/jj-and-novavax-data). In parallel, a number of potently neutralizing monoclonal antibodies (mAbs) have been developed that are in late-stage trials to be used prophylactically or therapeutically (Baum et al., 2020, Yang et al., 2020).

SARS-CoV-2 is a large positive-stranded RNA virus; the major virion surface glycoprotein is the trimeric spike that attaches the virus to host cells via the ACE2 receptor and, through a series of conformational changes, allows fusion of host and virion membranes releasing the virus RNA into the cell to start the infection cycle (Hoffmann et al., 2020; Ou et al., 2020). Spike is the target of RNA (Polack et al., 2020; Baden et al., 2021), viral vectored (Voysey et al., 2021), and inactivated virus and recombinant protein-based vaccines (Yadav et al., 2020).

Because of the huge number of genome replications that occur in infected populations and error-prone replication, viral mutations do and will continue to occur (Robson et al., 2020). Although the vast majority will be inconsequential or detrimental to viral fitness, a few may give the virus a competitive advantage and be the subject of rapid natural selection relating to transmission advantage, including enhanced replication and immune evasion. This leads to the emergence of dominant new variant viruses. Coronaviruses, as we are seeing with COVID-19, have the potential to alter their proteins with dramatic effect (Denison et al., 2011).

In recent months, a number of mutations in the spike protein have been exemplified by viruses that have grown in alternative hosts such as mink and transmitted back to humans or in immunocompromised chronically infected individuals (Kemp et al., 2020; Oude Munnink et al., 2021; Hayashi et al., 2020). While most of these mutations currently show little evidence of a selective advantage in humans, variants have been identified with multiple mutations in spike, which appear to have distinct selective advantages and have rapidly expanded in prevalence, notably that first identified in Kent in the UK (lineage B.1.1.7) and unrelated variants detected in South Africa (501Y.V2 also known as B.1.351) and Manaus in Brazil (P.1). All of these contain mutations in the ACE2 receptor binding footprint of the receptor binding domain (RBD), one in B.1.1.7, three in 501Y.V2, and three in P.1, with the N501Y mutation being common to all. It is believed that these mutations in the ACE2 receptor binding domain increase the affinity for ACE2 (Zahradník et al., 2021). These mutations also fall within the footprint of a number of potent neutralizing antibodies likely to afford vaccine-induced protection and of several candidate therapeutic mAbs (Cheng et al., 2021, Greaney et al., 2021; Nelson et al., 2021), thus potentially affording mutant viruses greater fitness to infect new hosts and also to escape from pre-existing antibody responses.

**Figure 1:**
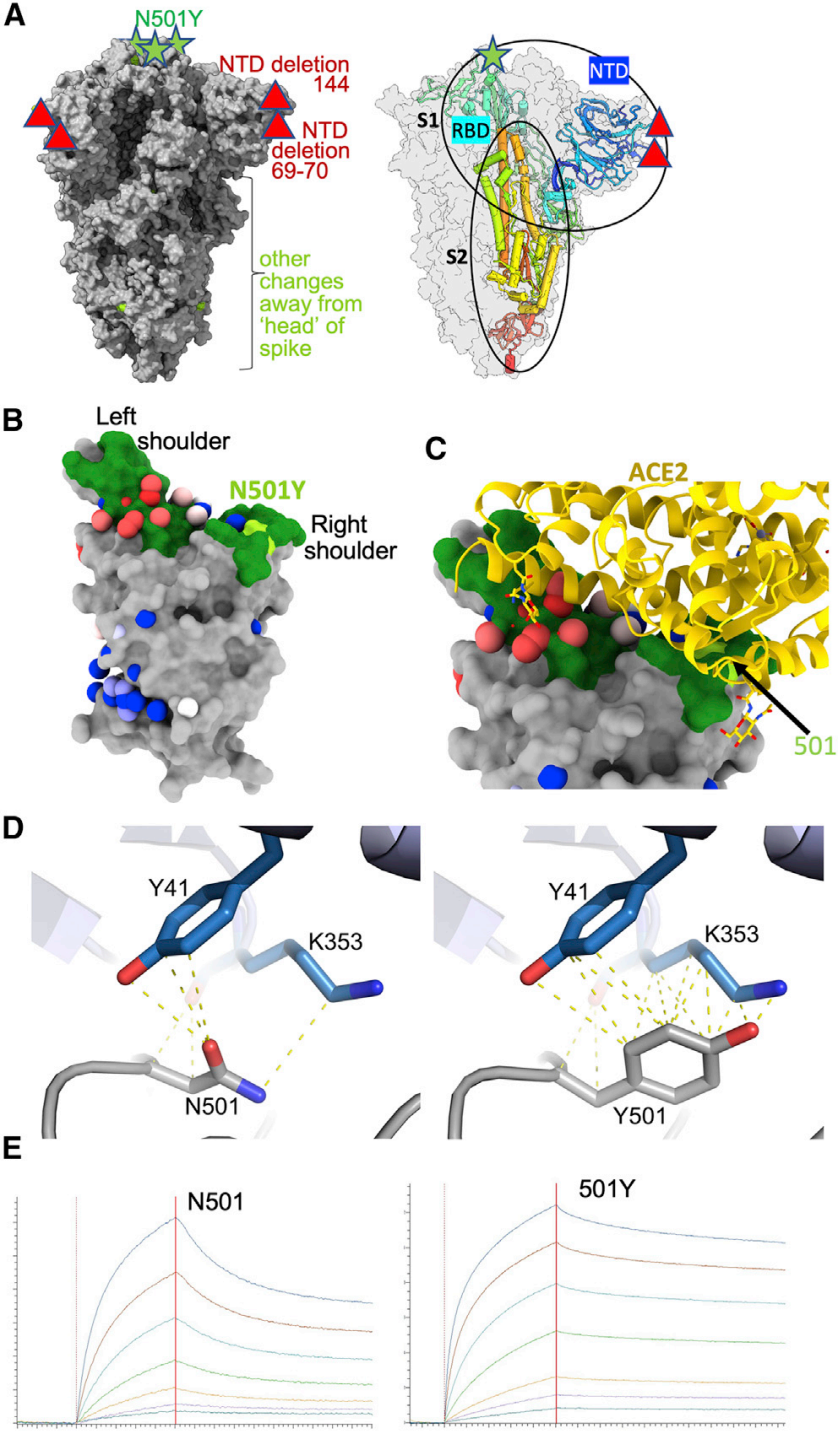
The B.1.1.7 variant spike protein and effect on ACE binding of the N501Y mutation (A) The SARS-CoV-2 spike trimer is depicted as a gray surface with mutations highlighted in yellow-green or with symbols. The RBD N501Y and the NTD 144 and 69–70 deletions are highlighted with green stars and red triangles, respectively. On the right, a protomer is highlighted as a colored ribbon within the transparent gray spike surface, illustrating its topology and marking key domains. (B) The RBD “torso” analogy. The RBD is represented as a gray surface with the ACE2 receptor binding site in dark green. Binding sites for the panel of antibodies (Dejnirattisai et al., 2021) on which this study draws are represented by spheres. The spheres represent the point at which placing spherical antibodies would optimally predict the BLI competition data and are colored according to their neutralization, from red (potent) to blue (non-neutralizing). The position of the B.1.1.7 N501Y mutation in the RBD is highlighted in light green toward the right shoulder. (C) Proximity of ACE2 to N501Y. The RBD is depicted as in (B) with ACE2 bound (in yellow cartoon format) with glycosylation drawn as sticks. (D) Left panel: interactions of N501 of WT RBD with residues Y41 and K353. The structure shown is the complex of N501 RBD with ACE2 determined by X-ray crystallography (PDB ID 6M0J, Lan et al., 2020). When the 501 is mutated to a tyrosine with the conformation seen in the N501Y RBD-269 Fab complex (right panel), Y501 makes T-shaped ring stacking interactions with Y41 and more hydrophobic contacts with K353 of ACE2 (note there are minor clashes of the side chain of Y501 to the end of the K353 side chain, which has ample room to adjust to optimize interactions). (E) BLI plots for WT (left) and N501Y (right) RBDs binding to ACE2. A titration series is shown for each (see STAR Methods). Note the much slower off rate for the mutant.

Such variants will continue to appear; indeed, global surveillance by sequencing of viral isolates is wholly inadequate, and many may already be present but undetected. The B.1.1.7 variant was first identified in a sequence taken from a patient at the end of September 2020 (Rambaut et al., 2020). The variant has rapidly become dominant in many areas of the UK, which has coincided with a rapid increase of infections during the second wave of the pandemic, with cases and hospitalizations in excess of those seen during the first phase. The B.1.1.7 variant is estimated to be 30%–60% more infectious than strains encountered in the first wave (Walker et al., 2021) and able to overcome public health efforts to contain infection. B.1.1.7 contains a total of 9 changes in the spike protein relative to Wuhan: N501Y, A570D, D614G, P681H, T716I, S982A, D1119H, and deletions of residues 69–70 and 144. Mutation N501Y perhaps gives the greatest concern as it has the potential to increase RBD/ACE2 affinity while also disrupting the binding of potent neutralizing antibodies (Figure 1A).

Here, we describe analysis of the cross-reactivity of the antibody responses to earlier SARS-CoV-2 viruses and the newly emerging B.1.1.7 variant. We take advantage of a previous study where we generated and analyzed 377 antibodies targeting the SARS-CoV-2 spike protein of which 80 target the RBD (Dejnirattisai et al., 2021); here, we analyze in detail the 20 most potent neutralizing antibodies (FRNT50 < 100 ng/mL), 19 anti-RBD, and 1 anti-NTD (N-terminal domain). A detailed structure-function analysis enables us to map these antibodies to the RBD structure and model the consequences of the N501Y mutation upon antibody and ACE2 binding. We also compare the neutralization of B.1.1.7 and Victoria (SARS-CoV-2/human/AUS/VIC01/2020), an early Wuhan-related strain of SARS-CoV-2 (Caly et al., 2020) viruses using mAbs, immune sera from infected cases and vaccinees, and examine neutralization of sera obtained from a small cohort of cases infected with the B.1.1.7 variant of SARS-CoV-2. In most cases, the mAbs showing reduced neutralization with B.1.1.7 are compatible with structural predictions, but in the case of mAb 269 crystal structures of the wild-type and B.1.1.7 RBD complexes provided an explanation of this sensitivity.

## Results

### The B.1.1.7 lineage

**Figure S1:**
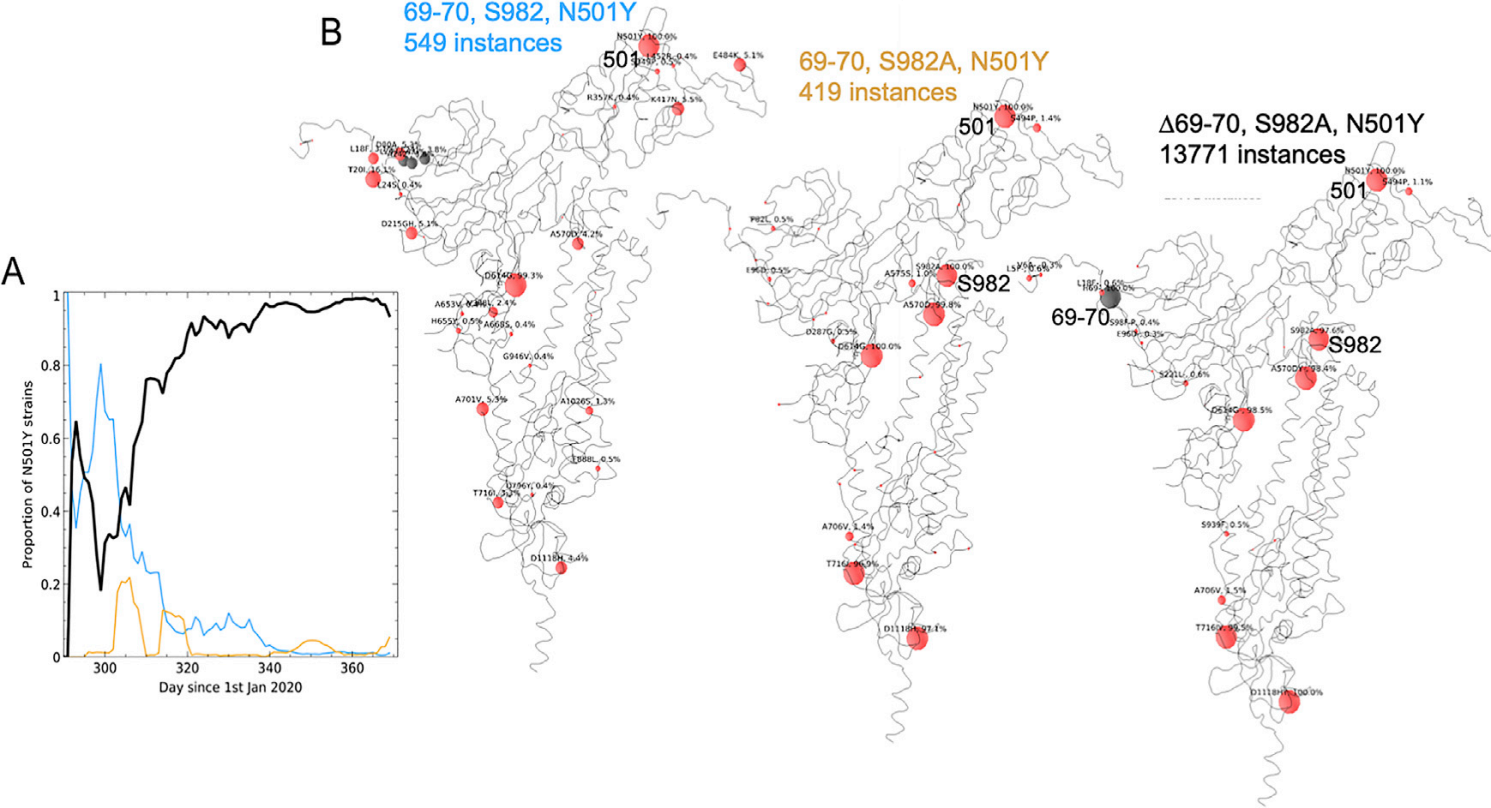
N5-1Y-containing sequences in the UK, related to STAR Methods (A) proportion of three subgroups of B.1.1.7 expressed as percentage of total 501Y-containing identifiable sequences. Black line shows dominant form with 501Y and Δ69-70. Blue, orange lines both lack 69-70 and have either wild-type or S982A mutation respectively. (B) associated mutations for blue (left), orange (middle) and black (right) plotted on Spike protein structure where modeled, with extended modeled N terminus (PDB: 6ZWV). Red circles show point mutations (circle size proportional to the log of the 100% occurrence), gray circles show deletions.

Analysis of 180,000 sequences from the COG-UK database (https://www.cogconsortium.uk) showed one major and two minor subgroups (Figure S1A) of the ∼13,700 identifiable variants harboring N501Y distinguishable by cluster analysis (STAR Methods). The major strain was the Δ69-70 B.1.1.7 strain, whereas the two minor groups lack this deletion and either had wild-type or the S982A mutation (Figure S1B). A mixture of all three subgroups at the outset in late October resolved into the dominance of the B.1.1.7 variant over the course of about 2 months (Figure S1A), suggesting the Δ69-70 mutation may be driving the evolutionary advantage over these two other forms. During the week of December 24, 2020, the N501Y variant represented 53% of the sequenced isolates in Great Britain.

### Characterizing the N501Y mutation in the RBD

The RBD may be likened to a classic human torso; in this analogy, the shoulders and neck are involved in interactions with the ACE2 receptor (Figures 1B and 1C) (Dejnirattisai et al., 2021). In this context, residue 501 lies within the footprint of the receptor on the right shoulder and is involved in hydrophobic interactions, especially with the side chains of residues Y41 and K353 of ACE2 with the 501 mutation from N to Y offering the opportunity for enhanced interactions (Figures 1C and 1D).

### Effect on ACE2 affinity

It has been reported that mutations at 501 can increase affinity for ACE2 (Starr et al., 2020; Gu et al., 2020), although these data are not for the mutation to Y. In contrast, Zahradník et al. (2021) report direct selection of N501Y when evolving the RBD to enhance affinity. We therefore investigated the effect of this mutation on ACE2 binding by RBD using biolayer interferometry (BLI) (Figure 1E). The results indicate a marked (7-fold) increase in binding affinity due to a slower off rate: WT RBD(501N)-ACE2: K_D_ 75.1 nM (K_on_ 3.88E4 /Ms, K_off_ 2.92E-3 /s), RBD(501Y)-ACE2: K_D_ 10.7 nM (K_on_ 6.38E4 /Ms, K_off_ 6.85E-4/s). This is in-line with enhanced interactions of the tyrosine side chain with the side chains of residues Y41 and K353 of ACE2 (Figure 1D). In the context of a multivalent interaction at the cell surface, this effect would be amplified. This alone might account for the selection of the N501Y mutation and an increase in transmission.

### Effect on mAb affinity

To investigate the effect of the N501Y mutation on antibody binding, we took advantage of our set of 377 mAbs (80 of which mapped to the RBD) generated from SARS-CoV-2 cases infected during the first wave of the pandemic in the UK using samples collected before June 2020 (Dejnirattisai et al., 2021). In that study, neutralization titers established that all 20 potent neutralizing antibodies (FRNT50 <0.1 μg/mL for the Victoria virus) bound the RBD, with the single exception of mAb 159, which bound the NTD. As expected, there was a correlation between ACE2 blocking and neutralization titers, and we solved structures of mAb immune complexes and used BLI competition experiments to impute and validate a detailed map of antibody binding across the majority of the RBD surface (Dejnirattisai et al., 2021). Potent mAbs mapped to positions either overlapping or closely adjacent to the ACE2 binding surface at the apex of the RBD (Figures 1B and 1C) and tended to have rather few somatic mutations (on average 5.33 and 4.33 amino acids in the heavy and light chains [HC, LC], respectively). A number of public antibody responses including IGHV3-53 (5 potent mAbs), IGHV1-58 (4 potent mAbs), and IGHV3-66 (2 potent mAbs) were present in the collection of RBD-specific mAbs and have been found in other studies (Barnes et al., 2020, Yuan et al., 2020; Liu et al., 2020).

**Figure 2:**
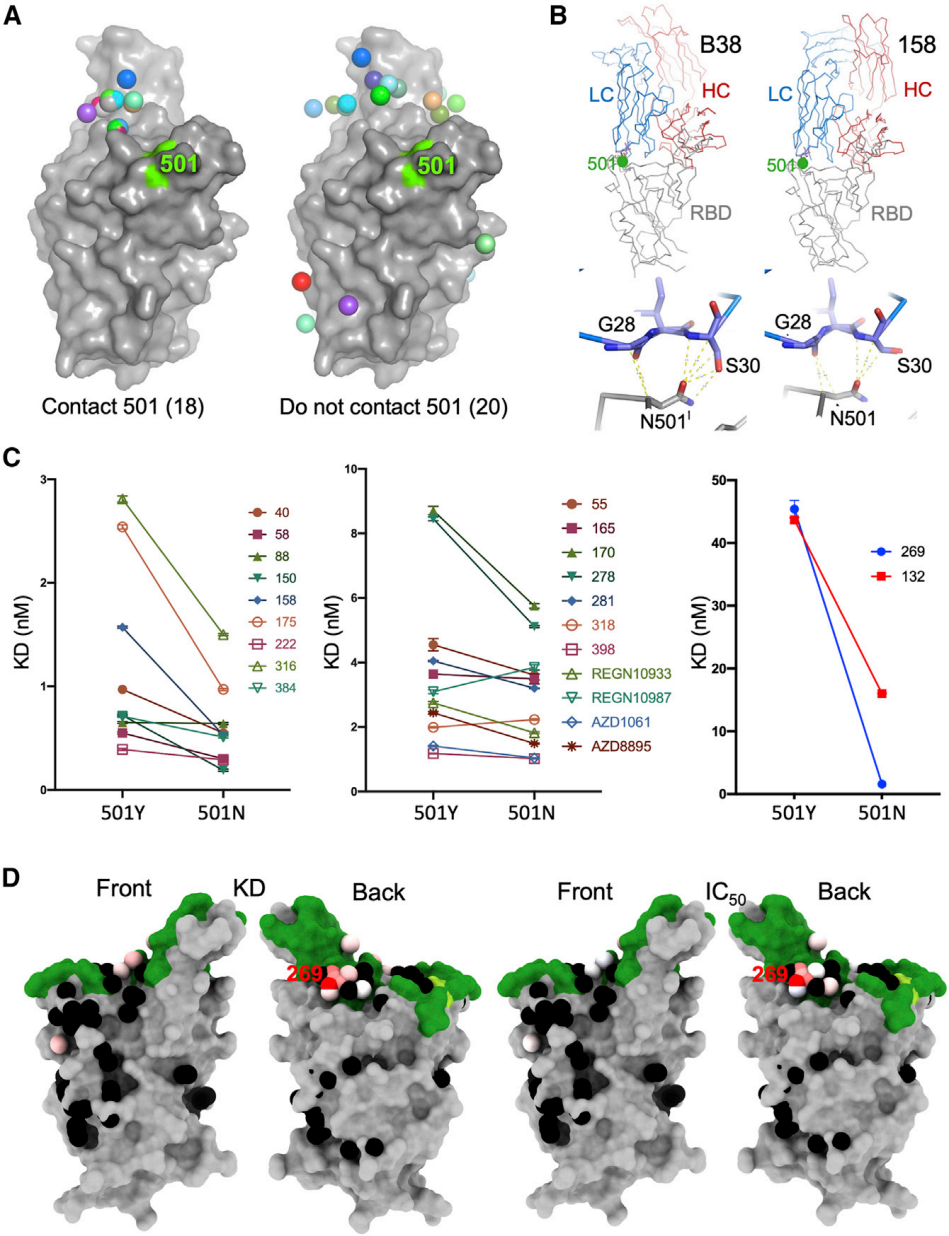
mAb binding to WT and N501Y RBD (A) Structural overlay of RBD-Fab complexes in which Fabs have direct contact with N501. The overlay was done by superimposing the RBD. Structures of 38 antibody Fabs in complex with RBD were analyzed. 18 have direct contact with N501 (left), which includes 14 IGHV3-53, 2 IGHV3-66 and two others. 20 Fabs do not have direct contact with N501 of the RBD (right), these include 3 IGHV3-53 or IGHV3-66 Fabs (Table S3). The RBD is shown as a gray surface with the ACE2 binding surface dark green and residue N501 highlighted in yellow-green. The Fabs are shown as spheres positioned at the tips of the CDR-H3s. (B) Examples of optimized binding to the asparagine 501 side chain for antibodies B38 (PDB ID 7BZ5) and 158 (PDB ID 7BEJ). (C) BLI results for potent binders selected from a panel of antibodies (Dejnirattisai et al., 2021) comparing 501Y RBD with 501N RBD. Error bars are derived from curve fitting and may underestimate experimental error. (D) Left pair: BLI data mapped onto the RBD using mabscape (https://github.com/helenginn/mabscape) and the method described in Dejnirattisai et al., 2021. The spheres represent the point at which placing spherical antibodies would optimally predict the BLI competition data. Front and back views of the RBD are depicted as in (A) but with the spheres representing the antibody binding sites colored according to the log of the ratio (K_D_501Y/K_D_501N). For white, the ratio is 1; for red, it is <0.1 (i.e., at least 10-fold reduction). Note the strong concordance between the two effects, with 269 being the most strongly affected. The nearby pink antibodies are mainly the IGHV3-53 and IGHV3-66 antibodies. See also Table S2.

Analysis of the position of the N501Y change with respect to the binding of all structurally characterized potent mAbs suggests that the binding of over half of the antibodies would be unaffected by the change (Figure 2A). However, one class of public antibodies have attracted particular attention, those using IGHV3-53 (Dejnirattisai et al., 2021; Yuan et al., 2020; Wu et al., 2020). For these and the IGHV3-66 antibodies, the mode of binding is dictated by the HC CDR1 and CDR2, which orientates the antibody such that the light-chain CDR1 region lies atop residue 501. We would expect the majority of these antibodies to be affected by the mutation, since, for them, unlike ACE2, the interaction with the asparagine is strongly favorable (Figure 2B).

**Figure 3:**
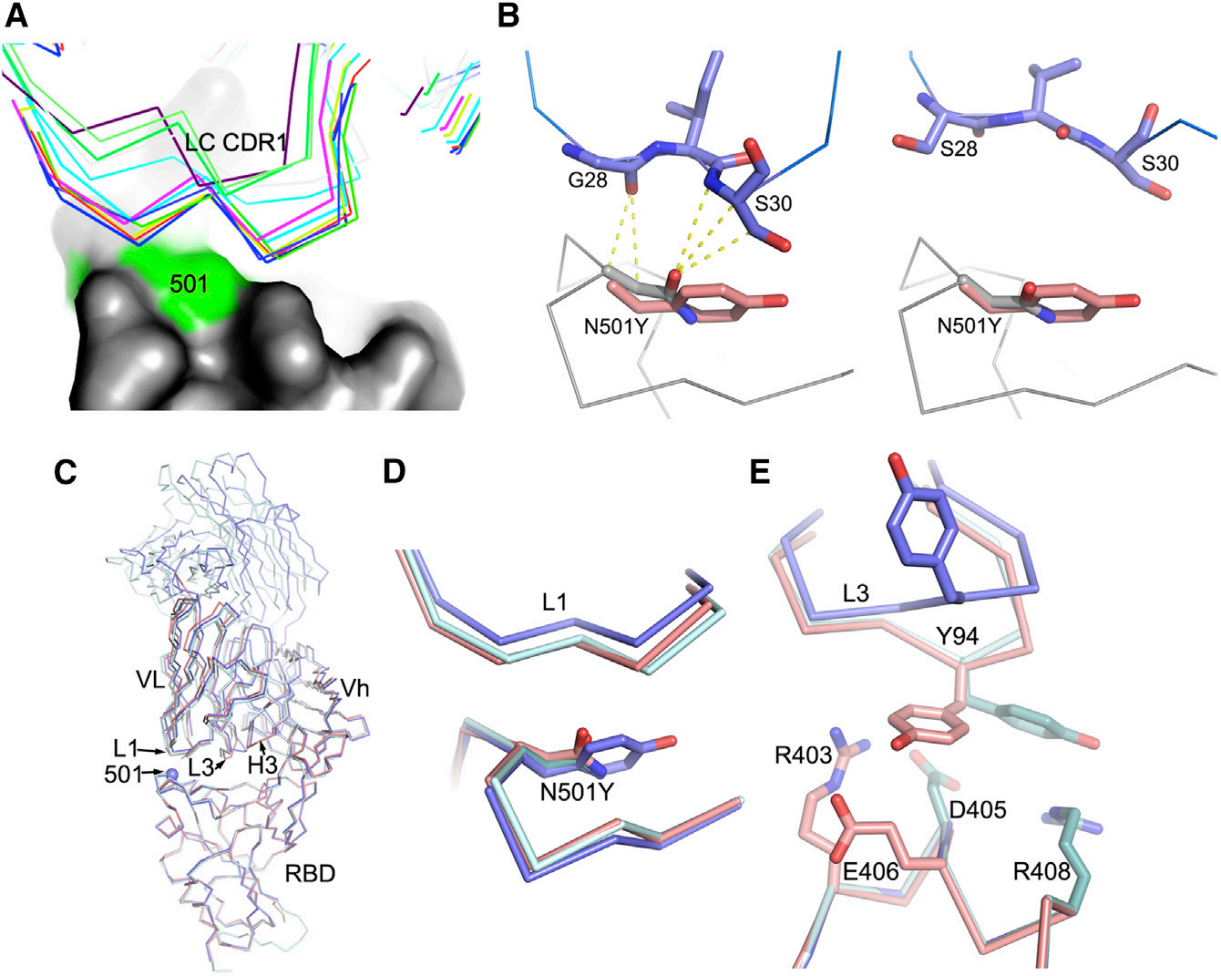
Molecular mechanisms of escape and comparison of N501Y RBD/269 Fab and RBD/scFv269 complexes (A) CDR-L1 (thin sticks) positions of a panel of V3-53 Fabs relative to N501 on the RBD (surface, with N501 highlighted in green). (B) The side chain of N501 makes extensive contacts with residues from CDR-L1 in the RBD-158 Fab complex (left, PDB: 7BEJ). In the right panel, N501 does not make any contact with p2c-2f11 Fab (PDB: 7CDI) whose LC is most similar in sequence and has the same CDR-L1, L2, and L3 lengths to mAb 222 shown by a blast of the LC of 222 against the PDB. The orientation and position of Y501 in the N501Y RBD/269 Fab complex is shown by overlapping the RBDs in both panels. (C) Crystallographic structures of RBD/Fab 269, N501Y RBD/Fab 269, and RBD/scFv269. Overlay of Cαs of N501Y RBD/Fab 269 (blue) with RBD/Fab 269 (cyan) and RBD/scFv269 (salmon) by superimposing the RBDs of the three complexes (PDB: 7NEG, 7NEH, 7BEM, respectively). (D) Structure changes in the 496–501 loop of the RBD and the CDR-L1 loop that contacts the mutation site. (E) Structural differences of the CDR-L3 loops between the three complexes. See also Tables S1 and S3.

**Figure S2:**
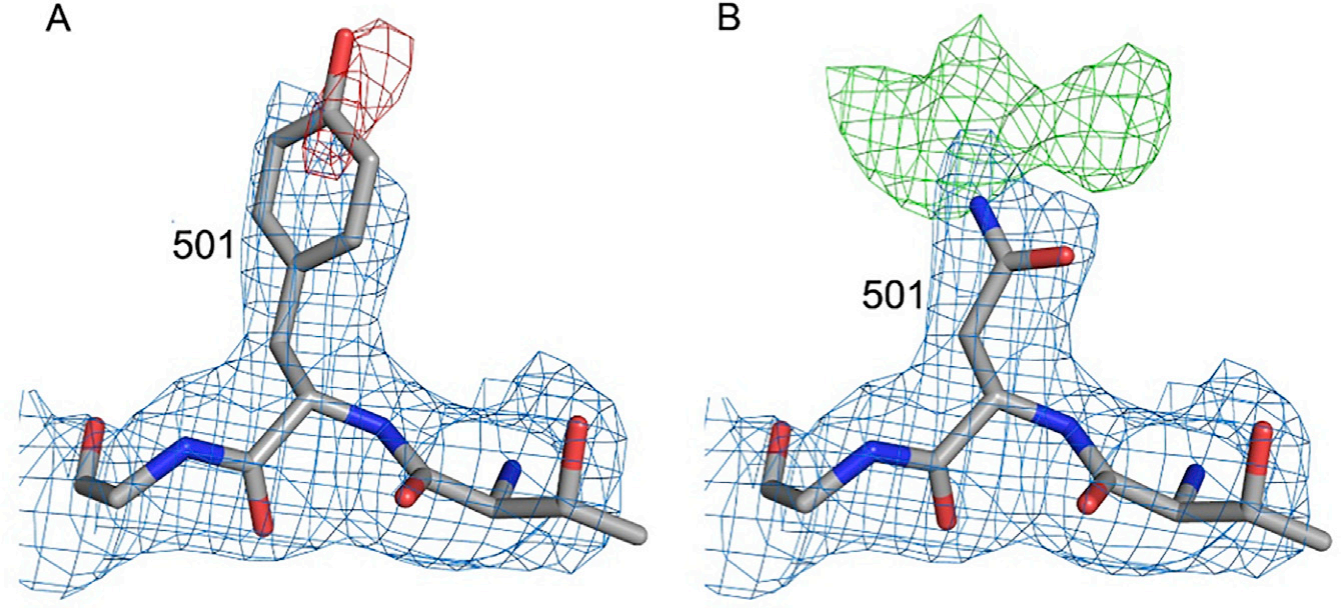
Electron density maps for residue 501, related to Figure 3 Electron density maps for RBD_N501Y_/Fab269 with residue 501 refined as a tyrosine in (A) and as an asparagine in (B). 2Fo-Fc maps are contoured at 1.2 σ and colored in blue in both panels. The negative density (red) in (A) is contoured at −3 σ, and the positive density (green) in (B) at 3 σ.

To examine the effects on antibody binding, we performed BLI experiments comparing the binding of potently neutralizing mAbs to RBDs containing 501Y and 501N (STAR Methods; Figure 2C). The results are mapped to the RBD in Figure 2D. As expected, there is little effect on many potent antibodies, for instance, the IGVH1-58 antibodies: 55, 165, 253, and 318. There is a marked ∼3-fold effect for mAb 40 (IGHV3-66) and for most of the important IGHV3-53 antibodies (150, 158, and 175). However, there is a correlation between the LC for the IGHV3-53 antibodies and the magnitude of the effect; thus, the common IGLV1-9 antibodies (mAbs 150 and 158) show a consistent reduction in affinity of roughly 3-fold (Figure 3A). In contrast, mAb 222, which pairs IGHV3-53 with IGLV3-20, shows no reduction. When modeled using the most similar light chain from the PDB, IGLV3-20 does not contact residue 501 (Figure 3B). However, a survey of the various structures determined shows that IGHV3-53 frequently pairs with IGLV3-20 and often results in 501 contacts. mAb 269, which is also a VH3-53 mAb, paired with the IGLV1-9 light chain, however, appears hyper-sensitive to the mutation (30-fold effect). The structure of Fab 269 in complex with WT RBD determined at 1.8 Å resolution (STAR Methods; Table S1; Figure 3C) shows similar interactions to those observed for mAbs 150 and 158. In order to understand this further, we determined the crystal structure of Fab 269 in complex with RBD harboring 501Y at 2.2 Å resolution (STAR Methods; Figure S2; Table S1). The result is shown in Figure 3C. The mutation introduces a rather small displacement of the L1 loop (Figure 3D), but there is a concomitant effect of the neighboring L3 loop (Figure 3E), with a significant switch in the position of Y94, abrogating contacts with residues R403 and E406 of the RBD. It is clear that further work is required to fully understand the sensitivity of antibodies binding in the IGHV3-53 mode to the N501Y mutation.

Finally, we looked at 2 sets of mAbs that have reached late-stage clinical trials for SARS-CoV-2: Regeneron REGN10933 and REGN10987 and AstraZeneca AZD1061 and AZD8895. Only a modest effect was observed with these antibodies (Figure 2C).

### Effect of B.1.1.7 mutations on neutralization by potent mAbs

**Figure 4:**
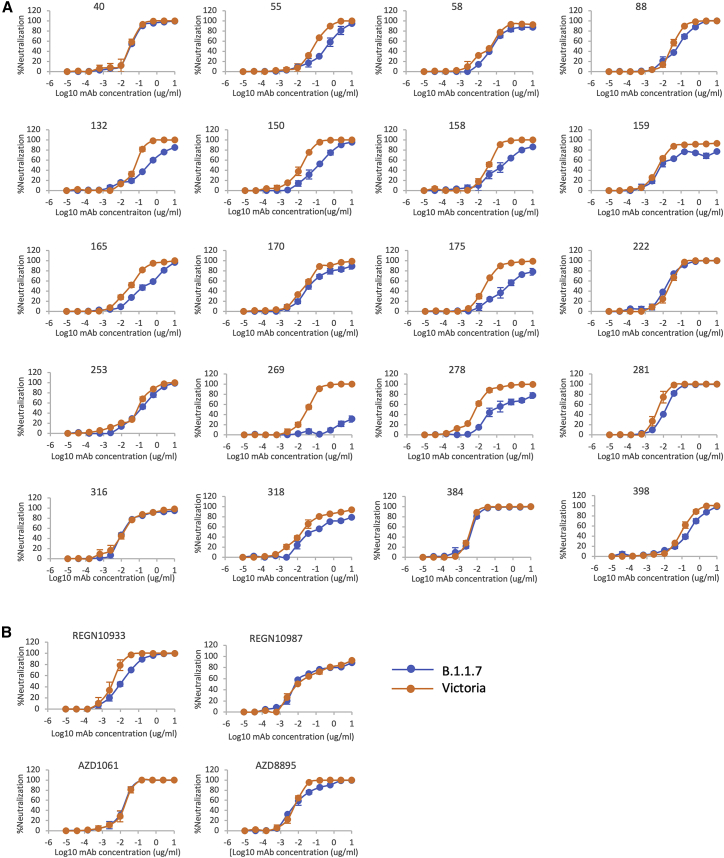
Neutralization of SARS-CoV-2 strains Victoria and B.1.1.7 by mAb (A) Neutralization curves of potent (FRNT50 <100 ng/mL) anti-RBD antibodies including those expressing the public heavy chain VH3-53 (150, 158, 175, 222, 269). (B) Regeneron antibodies, REGN10933 and REGN10987, and AstraZeneca antibodies, AZD8895 and AZD1061, are included for comparison. Neutralization of SARS-CoV-2 was measured using a focus reduction neutralization test (FRNT). Data are shown as mean ± SEM. See also Table S2.

Next, we performed neutralization assays with the potent mAb targeting the ACE2 interacting surface of RBD. Neutralizations were performed using focus reduction neutralization tests (FRNT) using viral strains Victoria and B.1.1.7 obtained from Public Health England (Figure 4A; Table S2). For some antibodies (40, 88, 222, 316, 384, 398), FRNT 50 values between B.1.1.7 and Victoria strains were minimally affected (<2-fold difference). However, for others there was a fall in the neutralization titers for B.1.1.7, particularly pronounced for mAb 269, where neutralization was almost completely lost and mAb 278, which failed to reach 100% neutralization showing a maximum of only 78%. For the Regeneron antibodies, the neutralization of REGN10987 was unaffected by B.1.1.7, while REGN10933 showed a slight reduction but still retained potent activity (Figure 4B; Table S2). The neutralization of the AZ antibodies AZD1061 and AZD8895 was similarly little affected.

### Neutralization activity of convalescent plasma and vaccine sera

**Figure 5:**
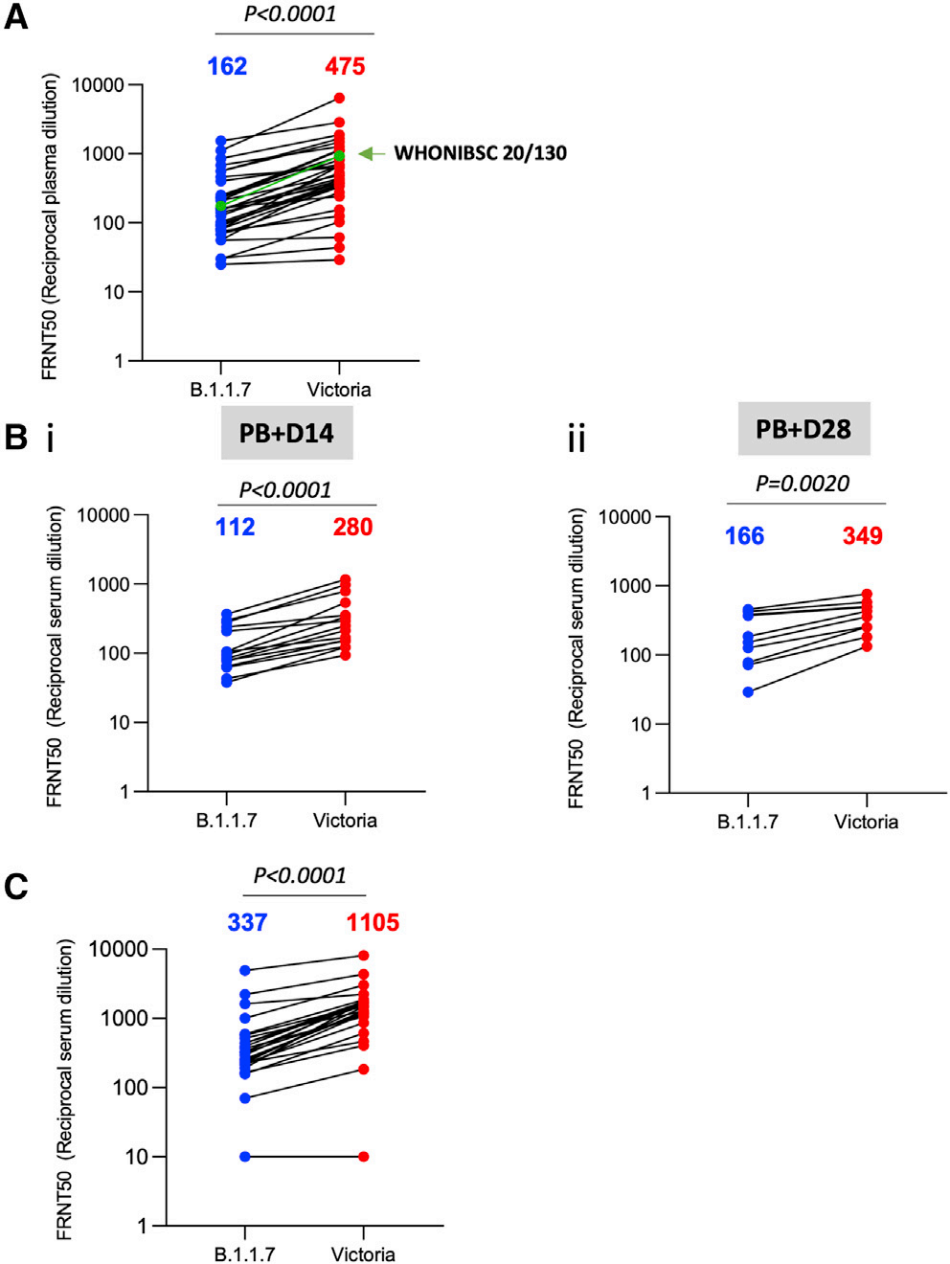
Neutralization activity of convalescent plasma and vaccine sera (A) Neutralization titers of 34 convalescent plasma samples collected 4–9 weeks following infection are shown with the WHONIBSC 20/130 reference serum (B) Neutralization titers of serum from volunteers vaccinated with the AstraZeneca vaccine ADZ1222, samples were taken at (1) 14 days following the second dose (n = 15) and (2) 28 days following the second dose (n = 10). (C) Neutralization titers of serum taken from volunteer healthcare workers recruited following vaccination with Pfizer-BioNTech BNT162b2 (n = 25). Neutralization was measured by FRNT, the Wilcoxon matched-pairs signed-rank test was used for the analysis, and two-tailed p values were calculated; geometric mean values are indicated above each column.

During the first wave of infection, before the emergence of B.1.1.7 strain, we collected a number of samples from cases at convalescence (4–9 weeks following infection) for the generation of mAbs. Stored plasma from these cases was used in neutralization assays comparing Victoria and B.1.1.7 (Figure 5A). We analyzed 34 convalescent samples including the WHONIBSC 20/130 reference serum, and, although a few sera showed near identical FRNT 50 values, the FRNT50 dilutions for the B.1.1.7 strain were 2.9-fold lower (geometric mean) than those for the Victoria strain (p < 0.0001).

We also assayed neutralization of the B.1.1.7 and Victoria strains using serum obtained from recipients of the Oxford-AstraZeneca and Pfizer vaccines. For the AstraZeneca AZD1222 vaccine, serum was obtained at baseline and at 14 and 28 days following the second dose. For the Pfizer vaccine, serum was obtained 7–17 days following the second dose of vaccine, which was administered 3 weeks after the first dose (participants were seronegative at entry). Neutralization assays against B.1.1.7 and Victoria strains showed a 2.5-fold (geometric mean n = 15, p < 0.0001) and 2.1-fold (geometric mean, n = 10, p < 0.002) reduction in the neutralization titers between B.1.1.7 and Victoria strains for the AstraZeneca vaccine after 14 and 28 days following the second dose, respectively (Figure 5B). For the Pfizer-BioNTech vaccine BNT162b2, the reduction was 3.3-fold (geometric mean, n = 25 p < 0.0001) (Figure 5C).

**Figure 6:**
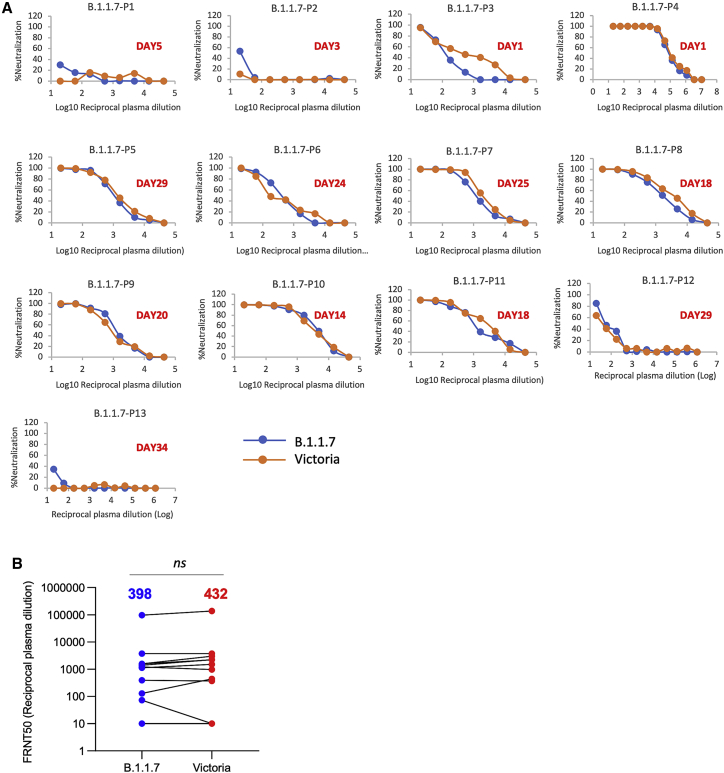
Neutralization activity of serum taken from patients suffering infection with B.1.1.7 (A) Neutralization titers of plasma from 13 patients infected with B.1.1.7 at various time points following infection. The days since infection are indicated in each panel. Neutralization was measured by FRNT. (B) Comparison of FRNT50 titers of individual sera against Victoria and B.1.1.7 strains, the number above each column is the geometric mean, the Wilcoxon matched-pairs signed-rank test was used for the analysis, and two-tailed p values were calculated.

Finally, we obtained plasma from 13 patients infected with B.1.1.7 (all had spike gene dropout in viral PCR testing and 11 were verified by sequencing) at various time points following infection and compared neutralization between B.1.1.7 and Victoria strains (Figure 6A). At early time points, neutralization titers were low or absent except in 1 case taken at day 1 of illness who showed identical neutralization of both viruses and was the highest titer of all the samples we have measured in this study; we speculate that this may represent a reinfection with B.1.1.7. For these samples as a whole, there was no significant difference between the neutralization titers for the two viruses (Figure 6B) meaning that infection with B.1.1.7 will afford protection from infection with earlier variants.

In conclusion, the neutralization assays on convalescent and vaccine serum revealed that the B.1.1.7 virus required higher concentrations of serum to achieve neutralization, although there was no evidence that the B.1.1.7 virus could evade neutralization by serum raised to early SARS-CoV-2 strains or vaccines.

## Discussion

We have shown here that neutralizing responses against the Victoria virus are less effective against B.1.1.7 and that part of this effect is due to the N501Y mutation as demonstrated by the weaker binding of a number of antibodies to the RBD, where N501Y is the only difference. The reduced binding and neutralization were particularly marked for some, but not all, members of the public IGHV3-53 class of mAb, since the IGHV3-53-defined CDR1 and CDR2 cause the antibody to orient the light chain in close proximity to Y501. However, B.1.1.7 contains additional mutations that may have a bearing on neutralization, in particular, the deletions at 69–70 and 144 in the NTD. NTD binding antibodies, which do not block interaction with ACE2, have been described by a number of groups to be able to neutralize SARS-CoV-2 (Chi et al., 2020; Liu et al., 2020; Cerutti et al., 2021, Suryadevara et al., 2021), with some antibodies showing IC_50_ values sub 10 ng/mL. In this study, B.1.1.7 showed only a 5.7-fold reduction in the FRNT50 for mAb 159 (FRNT50 Victoria 11 ng/mL B.1.1.7 61 ng/mL) suggesting that despite the residue 144 deletion being on the edge of the footprint for this antibody (Dejnirattisai et al., 2021) the binding site has not been completely disrupted.

The level of expression of ACE2 has been shown to correlate with likelihood of infection by SARS-CoV-1 (Jia et al., 2005), and the higher affinity for ACE2 of SARS-CoV-2 has been imputed to underlie its greater transmission. It is reasonable to assume that a further increase in affinity will increase the likelihood of the stochastic events of virus attachment resulting in localization for sufficient time to trigger, perhaps by the recruitment of additional receptors, internalization of the virus. As noted by Zahradník et al. (2021) in a situation where public health measures reduce R0 to below 1, there will be selective pressure to increase receptor affinity.

Here, we show that this increase in transmission is compounded by the reduction in neutralization potency of antibodies generated by prior infection. Modification of the ACE2 binding surface of the RBD would be predicted to directly disrupt the binding of antibodies that lose affinity to the mutated residues. However, antibodies that neutralize by ACE2 competition, even if not directly affected by the mutation, will have to compete with ACE2 for binding to the RBD, and mutations of RBD that increase the affinity of ACE2 will tip the equilibrium away from mAb/RBD interaction toward RBD/ACE2 making the virus more difficult to neutralize.

A mutation at 484 of the spike likely has a similar dual effect, and Zahradník et al. (2021) report that further affinity increase in ACE2 binding is possible. Although the most effort has been directed at generating antibodies that neutralize by blocking ACE2 binding, other mechanisms are possible (Huo et al., 2020; Zhou et al., 2020), and indeed partial or non-neutralizing antibodies may confer protection (Henry Dunand et al., 2016). Such antibodies would likely be unaffected by mutations in the ACE2 binding site, and they deserve more thorough investigation since they would form excellent components in therapeutic cocktails. In addition, natural exposure and vaccination may confer protective immunity against symptomatic and severe COVID-19 via memory T cell responses (Sariol and Perlman, 2020; Altmann and Boyton, 2020).

The recent description of a number of virus variants that appear to have developed independently is a cause for concern as it may signal the emergence of strains able to evade vaccine-induced antibody responses. There is now an imperative to closely survey the emergence of novel SARS-CoV-2 strains on a global basis and to quickly understand the consequences for immune escape. There is a need to define correlates of protection from SARS-CoV-2 and also to understand how T cells contribute to protection in addition to the antibody response. It is also imperative to understand whether the newly emerging strains including B.1.1.7, B.1.351, and P.1 are leading to more severe disease and whether they can evade natural or vaccine-induced immune responses (Wibmer et al., 2021, Zhu et al., 2021).

There is already work underway to modify vaccines directed toward viral variants, these modified vaccines can be built quickly to incorporate new strains, but they will be given to individuals with pre-existing immunity to ancestral strains, whether the modified vaccines will be able to effectively redirect the antibody response to the areas of difference in the novel strains rather than simply boosting the pre-existing response will need intensive study.

In summary, we describe here a modest reduction in the neutralization titers against B.1.1.7 by convalescent and vaccine sera generated to early strains of SARS-CoV-2 and give a structural/biophysical description of how this may be driven. However, although neutralization titers against B.1.1.7 are reduced, they remain robust, and there is no evidence of vaccine escape, which bodes well for protection against B.1.1.7 by the vaccines currently being deployed at massive scale against SARS-CoV-2.

### Limitations of the study

The correlates of protection from SARS-CoV-2 infection are yet to be established. The *in vitro* neutralization assays reported here do not convey the contributions to *in vivo* protection provided by T cells nor the contributions of Fcγ receptor interactions and complement activation. Convalescent plasma and vaccine serum were taken relatively soon after acute illness or following vaccination; it is possible that titers will drop over time to a point where they are no longer high enough to provide protection. It will be interesting to understand the antibody response made by people infected by B.1.1.7, particularly how antibodies adapt to the N501Y change, but also the deletions occurring in the NTD. It will also be instructive to look at how well convalescent or vaccine serum can neutralize the other recently described variants B.1.351 and P.1 and, conversely, how well serum from patients infected with these variants can neutralize B.1.1.7 and the original Wuhan strains.

## STAR★Methods

### Key resources table

**Table:** 

REAGENT or RESOURCE	SOURCE	IDENTIFIER
**Antibodies**		

Fab	Dejnirattisai et al., 2021	N/A
IgG	Dejnirattisai et al., 2021	N/A
Human anti-NP (mAb 206)	Dejnirattisai et al., 2021	N/A
Regeneron mAbs	AstraZeneca	Cat#REGN10933, REGN10987
AstraZeneca mAbs	AstraZeneca	Cat#AZD1061, AZD8895
Anti-Human IgG (Fc specific)-Peroxidase	Sigma	Cat#A0170; RRID:AB_257868

**Bacterial and virus strains**		

SARS-CoV-2 (Australia/VIC01/2020)	Caly et al., 2020	N/A
SARS-CoV-2/B.1.1.7	Public Health England	N/A
DH5α bacteria	*In Vitro*gen	Cat#18263012

**Biological samples**		

Serum from Pfizer-vaccinated individuals	University of Oxford	N/A
Serum from AstraZeneca-Oxford-vaccinated individuals	University of Oxford	N/A
Plasma from SARS-CoV-2 patients	John Radcliffe Hospital in Oxford UK	N/A

**Chemicals, peptides, and recombinant proteins**		

His-tagged SARS-CoV-2 RBD	This paper	N/A
His-tagged SARS-CoV-2 RBD N501Y	This paper	N/A
His-tagged human ACE2	This paper	N/A
Human ACE2-hIgG1Fc	This paper	N/A
Phosphate buffered saline tablets	Sigma-Aldrich	Cat#P4417
Dulbecco’s Modified Eagle Medium, high glucose	Sigma-Aldrich	Cat#D5796
Dulbecco’s Modified Eagle Medium, low glucose	Sigma-Aldrich	Cat#D6046
FreeStyle 293 Expression Medium	GIBCO	Cat#12338018
L-Glutamine–Penicillin–Streptomycin solution	Sigma-Aldrich	Cat#G1146
Fetal Bovine Serum	GIBCO	Cat#12676029
Polyethylenimine, branched	Sigma-Aldrich	Cat#408727
Carboxymethyl cellulose	Sigma	Cat#C4888
Strep-Tactin®XT	IBA Lifesciences	Cat#2-1206-025
HEPES	Melford	Cat#34587-39108
Sodium Chloride	Honeywell	Cat#SZBF3340H
LB broth	Fisher Scientific UK	Cat#51577-51656
Mem Neaa (100X)	GIBCO	Cat#2203945
Trypsin-EDTA	GIBCO	Cat#2259288
L-Glutamine 200 mM (100X)	GIBCO	Cat#2036885
SYPROorange (5000X in DMSO)	Thermo	Cat#S6651
Isopropyl β-d-1-thiogalactopyranoside	Meridian Bioscience	Cat#BIO-37036
Kanamycin	Melford	Cat#K22000
Lysozyme	Sigma-Aldrich	Cat#L6876
Tris-base	Melford	Cat#T60040
Imidazole	Sigma-Aldrich	Cat#56750
Triton X-100	Sigma-Aldrich	Cat#8787
Turbonuclease	Sigma-Aldrich	Cat#T4330
RNase A	QIAGEN	Cat#158922
NaCl	Sigma-Aldrich	Cat#S9888
MgSO4	Sigma-Aldrich	Cat#746452
Na2HPO4	Melford	Cat#S23100
NaH2PO4	Melford	Cat#S23185

**Deposited data**		

The coordinates and structure factors of the SARS-CoV-2 RBD-N501/Fab269 and SARS-CoV-2 RBD-501Y/Fab269 crystallographic complexes	This paper	PDB: 7NEH, 7NEG

**Experimental models: cell lines**		

HEK293S GnTI- cells	ATCC	Cat#CRL-3022; RRID:CVCL_A785
HEK293 cells	ATCC	Cat#CRL-3216
Expi293F Cells	GIBCO,	Cat#A14527; RRID:CVCL_0063
Hamster: ExpiCHO cells	Thermo Fisher	Cat#A29133
Vero cells	ATCC	Cat#CCL-81; RRID:CVCL_0059

**Recombinant DNA**		

Vector: pHLsec	Aricescu et al., 2006	N/A
Vector: pNEO	Aricescu et al., 2006	N/A
Vector: pOPING-ET	Nettleship et al., 2008	N/A
human ACE2 cDNA	Sourcebiosciences	Cat#5297380
Vector: human IgG1 heavy chain	German Cancer Research Center, Heidelberg, Germany (H. Wardemann)	N/A
Vector: human lambda light chain	German Cancer Research Center, Heidelberg, Germany (H. Wardemann)	N/A
Vector: human kappa light chain	German Cancer Research Center, Heidelberg, Germany (H. Wardemann)	N/A
Vector: Human Fab	Univeristy of Oxford	N/A
Vector: Human scFv	University of Oxford, NDM (G. Screaton)	N/A

**Software and algorithms**		

Xia2-dials	Winter et al., 2018	https://xia2.github.io/parameters.html
PHENIX	Liebschner et al., 2019	https://www.phenix-online.org/
COOT	Emsley and Cowtan, 2004	https://www2.mrc-lmb.cam.ac.uk/personal/pemsley/coot/
PyMOL	Warren DeLano and Sarina Bromberg	https://pymol.org/2/
Data Acquisition Software 11.1.0.11	Fortebio	https://www.sartorius.com/en/products/protein-analysis/octet-systems-software
Data Analysis Software HT 11.1.0.25	Fortebio	https://www.sartorius.com/en/products/protein-analysis/octet-systems-software
Prism 8.0	GraphPad	https://www.graphpad.com/scientific-software/prism/
IBM SPSS Software 26	IBM	https://www.ibm.com/us-en/?ar=1
mabscape	This paper	https://github.com/helenginn/mabscape
		https://snapcraft.io/mabscape

**Other**		

X-ray data were collected at beamline I03, Diamond Light Source, under proposal mx19946 for COVID-19 rapid access	This paper	https://www.diamond.ac.uk/covid-19/for-scientists/rapid-access.html
TALON Superflow Metal Affinity Resin	Clontech	Cat#635668
HiLoad 16/600 Superdex 200 pg	Cytiva	Cat#28-9893-35
Superdex 200 increase 10/300 GL column	Cytiva	Cat#28990944
HisTrap HP 5-ml column	Cytiva	Cat#17524802
HiTrap Heparin HT 5-ml column	Cytiva	Cat#17040703
Amine Reactive Second-Generation (AR2G) Biosensors	Fortebio	Cat#18-5092
Octet RED96e	Fortebio	https://www.sartorius.com/en/products/protein-analysis/octet-label-free-detection-systems
Buffer exchange system “QuixStand”	GE Healthcare	Cat#56-4107-78
Sonics vibra-cell vcx500 sonicator	VWR	Cat#432-0137
Cartesian dispensing system	Genomic solutions	Cat#MIC4000
Hydra-96	Robbins Scientific	Cat#Hydra-96
96-well crystallization plate	Greiner bio-one	Cat#E20113NN
Crystallization Imaging System	Formulatrix	Cat#RI-1000

### Resource availability

#### Lead contact

Resources, reagents and further information requirement should be forwarded to and will be responded by the Lead Contact, David I Stuart (dave@strubi.ox.ac.uk).

#### Materials availability

Reagents generated in this study are available from the Lead Contact with a completed Materials Transfer Agreement.

#### Data and code availability

The coordinates and structure factors of the SARS-CoV-2 RBD-N501/Fab269 and SARS-CoV-2 RBD-501Y/Fab269 crystallographic complexes are available from the PDB with accession codes PDB: 7NEH,7NEG, respectively. Mabscape is available from https://github.com/helenginn/mabscape, https://snapcraft.io/mabscape. The data that support the findings of this study are available from the corresponding authors on request.

### Experimental model and subject details

#### Serum from Pfizer vaccinated individuals

Pfizer vaccine serum was obtained 7-17 days following the second dose of vaccine which was administered 3 weeks after the first dose (participants were to the best of their knowledge seronegative at entry).

The study was approved by the Oxford Translational Gastrointestinal Unit GI Biobank Study 16/YH/0247 [research ethics committee (REC) at Yorkshire & The Humber – Sheffield]. The study was conducted according to the principles of the Declaration of Helsinki (2008) and the International Conference on Harmonization (ICH) Good Clinical Practice (GCP) guidelines. Written informed consent was obtained for all patients enrolled in the study. Vaccinees were Health Care Workers, based at Oxford University Hospitals NHS Foundation Trust, not known to have prior infection with SARS-C0V-2. Each received two doses of COVID-19 mRNA Vaccine BNT162b2, 30 μg, administered intramuscularly after dilution as a series of two doses (0.3 mL each) 18-28 days apart. The mean age of vaccinees was 43 years (range 25-63), 11 male and 14 female.

#### Serum from AstraZeneca-Oxford vaccinated individuals

Full details of the randomized controlled trial of ChAdOx1 nCoV-19 (AZD1222), were previously published (PMID: 33220855/PMID: 32702298). These studies were registered at ISRCTN (15281137 and 89951424) and ClinicalTrials.gov (NCT04324606 and NCT04400838). Written informed consent was obtained from all participants, and the trial is being done in accordance with the principles of the Declaration of Helsinki and Good Clinical Practice. The studies were sponsored by the University of Oxford (Oxford, UK) and approval obtained from a national ethics committee (South Central Berkshire Research Ethics Committee, reference 20/SC/0145 and 20/SC/0179) and a regulatory agency in the United Kingdom (the Medicines and Healthcare Products Regulatory Agency). An independent DSMB reviewed all interim safety reports. A copy of the protocols was included in previous publications (PMID: 33220855/PMID: 32702298).

Data from vaccinated volunteers who received two vaccinations are included in this paper. Vaccine doses were either 5 × 10^10^ viral particles (standard dose; SD/SD cohort n = 21) or half dose as their first dose (low dose) and a standard dose as their second dose (LD/SD cohort n = 4). The interval between first and second doses was in the range of 8-14 weeks. Blood samples were collected and serum separated on the day of vaccination and on pre-specified days after vaccination e.g., 14 and 28 days after boost.

#### Serum from infected individuals

Plasma and peripheral blood mononuclear cells were collected from individual with SARS-CoV2 confirmed through a test positive for SARS-CoV-2 using reverse transcriptase polymerase chain reaction (RT-PCR) from an upper respiratory tract (nose/throat) swab tested in accredited laboratories. Patients were recruited through a variety of studies including Sepsis Immunomics [Oxford REC C, reference:19/SC/0296]), ISARIC/WHO Clinical Characterization Protocol for Severe Emerging Infections [Oxford REC C, reference 13/SC/0149] and the Gastro-intestinal illness in Oxford: COVID substudy [Sheffield REC, reference: 16/YH/0247]. In all cases it was known whether a patient was recruited into one or several studies and clinical information including severity of disease (mild, severe or critical infection according to recommendations from the World Health Organization) and times between symptom onset and sampling and age of participant was known.

#### Bacterial strains and cell culture

Vero (ATCC CCL-81) cells were cultured at 37°C in Dulbecco’s Modified Eagle medium (DMEM) high glucose (Sigma) supplemented with 10% fetal bovine serum (FBS), 2 mM GlutaMAX (GIBCO, 35050061) and 100 U/ml of penicillin–streptomycin. Human mAbs were expressed in HEK293T cells cultured in UltraDOMA PF Protein-free Medium (Cat# 12-727F, LONZA) at 37°C with 5% CO2. *Ecoli DH5α* bacteria were used for transformation of plasmid pNEO-RBD N501Y. A single colony was picked and cultured in LB broth with 50 μg mL^-1^ Kanamycin at 37°C at 200 rpm in a shaker overnight. HEK293T (ATCC CRL-11268) cells were cultured in DMEM high glucose (Sigma) supplemented with 10% FBS, 1% 100X Mem Neaa (GIBCO) and 1% 100X L-Glutamine (GIBCO) at 37°C with 5% CO_2_. To express RBD, RBD N501Y and ACE2, HEK293T cells were cultured in DMEM high glucose (Sigma) supplemented with 2% FBS, 1% 100X Mem Neaa and 1% 100X L-Glutamine at 37°C for transfection.

#### Viral stocks

SARS-CoV-2/human/AUS/VIC01/2020 (Caly et al., 2020) and SAR-CoV-2/B.1.1.7, provided by Public Health England, were grown in Vero (ATCC CCL-81) cells. Cells were infected with the SARS-CoV-2 virus at multiplicity of infection of 0.0001. Virus-containing supernatant was harvested when 80% CPE was observed and spun at 2000 rpm at 4°C before being stored at −80°C. Viral titers were determined by a focus-forming assay on Vero cells. Both Victoria passage 5 and B.1.1.7 stocks passage 2, were sequence verified to contain the expected spike protein sequence and no changes to the furin cleavage sites.

### Method details

#### COG-UK sequence analysis

All COG-UK sequences were downloaded on 24^th^ January 2020, and the translated protein sequences were roughly to the wild-type reference from start and stop codons between nucleotides 21000-25000, and filtered on the mutation 501Y. Sequence alignment was carried out, and identified mutations were plotted as red balls (single point mutations) or black balls (deletions) on the modeled C-alpha positions of the spike structure, size proportional to the logarithm of the incidence. Residues which mutated at an incidence greater than 0.3% compared to the wild-type were labeled explicitly.

#### Cloning of native RBD, RBD N501Y and ACE2

The constructs of native RBD and ACE2 are the same as in Zhou et al. (2020). To clone RBD N501Y, a construct of native RBD was used as the template and two primers of RBD (Forward primer 5′-CTACGGCTTTCAGCCCACATACGGTGTGGGCTACCAGCCTT-3′ and reverse primer 5′-AAGGCTGGTAGCCCACACCGTATGTGGGCTGAAAGCCGTAG-3′) and two primers of pNEO vector (Forward primer 5′- CAGCTCCTGGGCAACGTGCT-3′ and reverse primer 5′- CGTAAAAGGAGCAACATAG-3′) were used to do PCR. Amplified DNA fragments were digested with restriction enzymes AgeI and KpnI and then ligated with digested pNEO vector. This construct encodes exactly the same protein as native RBD except the N501Y mutation, as confirmed by sequencing.

#### Protein production

Protein expression and purification were performed as described in Zhou et al. (2020) and Dejnirattisai et al., 2021. Briefly RBD and mAb were expressed in 293T cells, His-tagged RBD was purified on Ni-NTA and mAb on protein-A. The Regeneron and AstraZeneca antibodies were supplied by AstraZeneca.

#### Preparation of 269 Fab

Fab fragments of 269 antibody were digested and purified using Pierce Fab Preparation Kit, following the manufacturer’s protocol.

#### Bio-layer interferometry

BLI experiments were run on an Octet Red 96e machine (Fortebio). To measure the binding affinities of monoclonal antibodies with native RBD and RBD N501Y, RBD and RBD N501Y were immobilized onto AR2G biosensors (Fortebio) separately. Monoclonal antibodies were used as analytes. To measure the binding affinities of native RBD and RBD N501Y with ACE2, native RBD and RBD N501Y were immobilized onto AR2G biosensors separately. Serial dilutions of ACE2 were used as analytes. Data were recorded using software Data Acquisition 11.1 (Fortebio) and analyzed using software Data Analysis HT 11.1 (Fortebio) with a 1:1 fitting model.

#### Crystallization

269 Fab was mixed with RBD or N501Y RBD in a 1:1 molar ratio with a final concentration of 9.9 mg ml^−1^. After incubation at room temperature for 30 min, the sample was used for initial screening of crystals in Crystalquick 96-well X plates (Greiner Bio-One) with a Cartesian Robot using the nanoliter sitting-drop vapor-diffusion method as previously described (Walter et al., 2003). Crystals of RBD/269 Fab complex were grown in Molecular Dimensions Morpheus screen, condition C6 containing 0.09 M NPS (NaN03; Na2HPO4; (NH4)2SO4), 0.1 M buffer 2 (sodium HEPES, MOPS) and 30% EDO_P8K (ethylene glycol, PEG 8K). Crystals of N501Y RBD/269 Fab complex were obtained from a Molecular Dimensions Proplex screen, condition B10 containing 0.15 M ammonium sulfate, 0.1 M MES pH 6.0 and 15% PEG 4000.

#### X-ray data collection, structure determination and refinement

Crystals of N501 RBD/269 Fab were mounted in loops and dipped in solution containing 25% glycerol and 75% mother liquor for a second before being frozen in liquid nitrogen prior to data collection. No cryo-protectant was used for RBD/269 crystals. Diffraction data were collected at 100 K at beamline I03 of Diamond Light Source, UK. Diffraction images of 0.1° rotation were recorded on an Eiger2 XE 16M detector (exposure time of either 0.003 or 0.007 s per image, beam size 80 × 20 μm, 100% beam transmission and wavelength of 0.9763 Å). Data were indexed, integrated and scaled with the automated data processing program Xia2-dials (Winter, 2010; Winter et al., 2018). A dataset of 720° was collected from 2 frozen crystals to 2.19 Å resolution for N501Y RBD/269 Fab complex. 360° of data were collected for the RBD/269 Fab complex from a single crystal to 1.77 Å resolution.

Both structures were determined by molecular replacement with PHASER (McCoy et al., 2007) using search models of SARS-CoV-2 RBD/scFv269 complex (PDB: 7BEM) and the ChCl domains of SARS-CoV-2 RBD/158 complex (PDB: 7BEK) (Dejnirattisai et al., 2021). Cyclic model rebuilding with COOT (Emsley and Cowtan, 2004) and refinement with PHENIX (Liebschner et al., 2019) resulted in the current structures with R_work_/ R_free_ = 0.197/ 0.222 and R_work_/ R_free_ = 0.185/0.200 for all data to 2.19 Å and 1.77 Å resolution for N501Y RBD/269 Fab and RBD/269 Fab complexes, respectively. Electron density for the side chain of Y501 is weak. However, when the structure was refined with an asparagine at 501, there was strong, dispersed positive density around the side chain, consistent with the presence of a flexible tyrosine residue (Figure S2). Mass spectrometry and biolayer interferometry data confirmed the presence of tyrosine at 501. Data collection and structure refinement statistics are given in Table S1. Structural comparisons used SHP (Stuart et al., 1979), residues forming the RBD/Fab interface were identified with PISA (Krissinel and Henrick, 2007) and figures were prepared with PyMOL (The PyMOL Molecular Graphics System, Version 1.2r3pre, Schrödinger, LLC).

#### Focus reduction neutralization assay (FRNT)

The neutralization potential of an antibody was measured using a Focus Reduction Neutralization Test (FRNT), where the reduction in the number of the infected foci is compared to a no antibody negative control well. Briefly, serially diluted Ab or plasma was mixed with SARS-CoV-2 strain Victoria or B.1.1.7 and incubated for 1 hr at 37°C. The mixtures were then transferred to **96**-**well**, **cell culture-treated, flat-bottom microplate containing confluent Vero** cell monolayers in duplicate and incubated for further 2 hr, followed by the addition of 1.5% semi-solid carboxymethyl cellulose (CMC) overlay medium to each well to limit virus diffusion. A focus forming assay was then performed by staining Vero cells with human anti-NP mAb (mAb206) followed by peroxidase-conjugated goat anti-human IgG (A0170; Sigma). Finally, the foci (infected cells) approximately 100 per well in the absence of antibodies, were visualized by adding TrueBlue Peroxidase Substrate. **Virus-infected cell foci were counted on the classic AID EliSpot reader using AID ELISpot software.** The percentage of focus reduction was calculated and IC_50_ was determined using the probit program from the SPSS package.

### Quantification and statistical analysis

Statistical analyses are reported in the results and figure legends. Neutralization was measured by FRNT. The percentage of focus reduction was calculated and IC_50_ was determined using the probit program from the SPSS package.The Wilcoxon matched-pairs signed rank test was used for the analysis and two-tailed P values were calculated and geometric mean values. BLI data were analyzed using Data Analysis HT 11.1 (Fortebio) with a 1:1 fitting model.
