# Supplementary material for: Reduced neutralization of SARS-CoV-2 B.1.1.7 variant by convalescent and vaccine sera
Source: Cell. 2021 Apr 15;184(8):2201–2211.e7. doi: 10.1016/j.cell.2021.02.033 (PMC7891044; doi:10.1016/j.cell.2021.02.033)
Supplement: Document S1. Tables S1–S3 [file mmc1.pdf]

Cell, Volume *184*

Supplemental information

Reduced neutralization of SARS-CoV-2

B.1.1.7 variant by convalescent and vaccine sera

Piyada Supasa, Daming Zhou, Wanwisa Dejnirattisai, Chang Liu, Alexander J. Mentzer, Helen M. Ginn, Yuguang Zhao, Helen M.E. Duyvesteyn, Rungtiwa Nutalai, Aekkachai Tuekprakhon, Beibei Wang, Guido C. Paesen, Jose Slon-Campos, César López-Camacho, Bassam Hallis, Naomi Coombes, Kevin R. Bewley, Sue Charlton, Thomas S. Walter, Eleanor Barnes, Susanna J. Dunachie, Donal Skelly, Sheila F. Lumley, Natalie Baker, Imam Shaik, Holly E. Humphries, Kerry Godwin, Nick Gent, Alex Sienkiewicz, Christina Dold, Robert Levin, Tao Dong, Andrew J. Pollard, Julian C. Knight, Paul Klenerman, Derrick Crook, Teresa Lambe, Elizabeth Clutterbuck, Sagida Bibi, Amy Flaxman, Mustapha Bittaye, Sandra Belij-Rammerstorfer, Sarah Gilbert, David R. Hall, Mark A. Williams, Neil G. Paterson, William James, Miles W. Carroll, Elizabeth E. Fry, Juthathip Mongkolsapaya, Jingshan Ren, David I. Stuart, and Gavin R. Screaton

Table S1 X-ray data collection and refinement statistics. Related to STAR Methods X-ray data collection, structure determination and refinement

**Data collection**		
Data set	RBD/Fab269	RBD_{N501Y}/Fab269
Space group	$C222_1$	C2
Cell dimensions (Å)		
a, b, c (Å)	82.8, 149.7, 145.9	195.1, 85.0, 57.9
a, b, g (°)	90, 90, 90	90, 100.6, 90
Resolution (Å)	75–1.77 (1.80–1.77)	96–2.19 (2.23–2.19)
Unique reflections	88349 (4395)	48007 (2371)
R_{merge}	0.091 (---)	0.177 (---)
R_{pim}	0.025 (0.980)	0.050 (0.988)
$CC_{1/2}$	1.00 (0.333)	0.997 (0.468)
<I> /< σI>	14.0 (0.3)	8.4 (0.2)
Completeness (%)	100 (95.5)	99.9 (98.5)
Redundancy	13.5 (13.9)	13.0 (9.1)
**Refinement**		
Resolution (Å)	42.7–1.77	56.9–2.19
No. reflections	83704/4325	41325/2137
R_{work} /R_{free}	0.182/0.198	0.197/0.222
No. atoms	5454	4948
Average B-factors ($Å^2$)	50	68
R.m.s. deviations		
Bond lengths (Å)	0.009	0.003
Bond angles (°)	1.0	0.6
Ramachandran plot (%)		
Favoured	96.9	96.2
Allowed	3.1	3.8
Outliers	0	0

Numbers in brackets refer to the highest resolution shell of data.

Table S2. Neutralization and RDB binding of selected antibodies against SARS-CoV-2 strains Victoria and B.1.1.7. Related to STAR Methods Bio-Layer Interferometry and Focus Reduction Neutralization Assay, and Figures 2 and 4.

mAb	IC50 (ug/ml)#			KD (nM)€		Immunoglobulin gene usage∆		
	B.1.1.7	Victoria	B.1.1.7/Victoria ratio$	RBD-N501Y	Native RBD	IGHV	K/λ	IGLV
40	0.035 ± 0.008	0.026 ± 0.007	1.36	0.97±0.006	0.55±0.003	3-66	K	1-33 or 1D-33
55	0.348 ± 0.044	0.095 ± 0.015	3.68	4.55±0.19	3.61±0.15	1-58	K	3-20
58	0.116 ± 0.029	0.041 ± 0.003	2.81	0.55±0.004	0.30±0.003	3-9	λ	3-21
88	0.058 ± 0.008	0.033 ± 0.001	1.78	0.65±0.005	0.64±0.004	4-61	λ	1-36
132	0.337 ± 0.048	0.048 ± 0.000	7.03	43.7±0.43	16.0±0.09	4-34	λ	7-46
150	0.139 ± 0.019	0.012 ± 0.000	11.96	0.72±0.004	0.19±0.002	3-53	K	1-9
158	0.254 ± 0.109	0.031 ± 0.004	8.33	1.57±0.01	0.53±0.0003	3-53	K	1-9
159	0.061 ± 0.020	0.011 ± 0.000	5.71	N/A	N/A	3-30	K	3-20
165	0.212 ± 0.004	0.034 ± 0.004	6.31	3.64±0.14	3.49±0.15	1-58	K	3-20
170	0.105 ± 0.050	0.025 ± 0.004	4.22	8.71±0.13	5.75±0.07	5-51	K	2D-29
175	0.575 ± 0.280	0.026 ± 0.000	22.51	2.54±0.02	0.97±0.005	3-53	K	1-33 or 1D-33
222	0.014 ± 0.002	0.019 ± 0.000	0.74	0.39±0.005	0.29±0.003	3-53	K	3-20
253	0.126 ± 0.018	0.055 ± 0.008	2.28	19.5±0.20	13.9±0.48	1-58	K	3-20
269	>10	0.030 ± 0.000	N/A	45.4±1.42	1.59±0.009	3-53	K	1-9
278	0.307 ± 0.149	0.014 ± 0.007	22.46	8.45±0.06	5.11±0.03	1-18	K	1-39 or 1D-39
281	0.012 ± 0.000	0.005 ± 0.001	2.50	4.05±0.02	3.20±0.01	3-7	K	2-24
316	0.024 ± 0.005	0.018 ± 0.007	1.37	2.81±0.03	1.50±0.01	1-2	λ	2-8
318	0.185 ± 0.037	0.029 ± 0.008	6.30	1.99±0.02	2.23±0.02	1-58	K	3-20
384	0.005 ± 0.002	0.004 ± 0.001	1.13	0.71±0.006	0.51±0.004	3-11	K	1-27
398	0.180 ± 0.001	0.091 ± 0.004	1.98	1.18±0.009	1.02±0.007	3-66	λ	2-8
REGN10933	0.014 ± 0.002	0.004 ± 0.002	3.31	2.75±0.04	1.82±0.03			
REGN10987	0.028 ± 0.003	0.032 ± 0.007	0.86	3.09±0.06	3.85±0.08			
AZD1061	0.012 ± 0.002	0.013 ± 0.003	0.93	1.41±0.02	1.04±0.01			
AZD8895	0.011 ± 0.002	0.005 ± 0.001	2.17	2.44±0.04	1.49±0.02			

Neutralization activity of selected antibodies against SARS-CoV-2 strain Victoria and B.1.1.7 were determined by FRNT. Data are from 2 independent experiments, each with duplicate wells and the data are shown as mean ± s.e.m.

$ IC50 ratio between strains B.1.1.7 and Victoria.

€ KD values for Fab association with N501-RBD and Y501-RBD measured by BLI are shown mAb 159 binds to the NTD and was not tested.

∆ VH and VL gene usage for monoclonal antibodies.

Table S2

Table S3 RBD/Fab complexes analysed for N501 contact. Related to Figures 2 and 3.

**Fabs contact N501 (≤ 4 Å)**		**Fabs that do not contact N501 (>4 Å)**	
**Name**	**PDB ID**	**Name**	**PDB ID**
B38	7BZ5	CV07-270	6XKP
CC12.1	6XC3	P2B-2F6	7BWJ
CC12.3	6XC4	P2C-1A3	7CDJ
BD604	7CH4	BD368	7CHE
BD629	7CH5	P17	7CWO
BD236	7CHB	COVA2	7JMP
COVA2-04	7JMO	COVA1	7JMW
C102	7K8M	52	7KZ9
C1A-B12	7KFV	298	7KZ9
C1A-B3	7KFW	CR3022	6YLA
C1A-C2	7KFX	REGN10933	6XDG
C1A-F10	7KFY	REGN10987	6XDG
STE90-C11	7B3O	CV30	6XE1
Fab-7CJF	7CJF	CB6	7C01
40	7ND3	P2C-1F11	7CDI
150	7ND5	EY6A	6ZCZ
158	7ND6	S309	7BEP
269	7NEH	88	7BEL
		253	7BEN
		384	7BEP
